# Tankyrases and Their Binding Proteins: Origins of Their Roles in Diverse Cellular Pathways

**DOI:** 10.3390/cells15040348

**Published:** 2026-02-14

**Authors:** Nafiseh Chalabi Hagkarim, Roger J. Grand

**Affiliations:** 1 Department of Surgery & Cancer, Imperial College London, Hammersmith Campus, Du Cane Road, London W12 0NN, UK; 2 Department of Cardiovascular Medicine, The Medical School, University of Birmingham, Birmingham B15 2TT, UK

**Keywords:** tankyrase1, tankyrase2, TNKS, PARP5a/b, PARylation, RNF146, ubiquitylation, protein degradation, TBM domain, signaling, Wnt/β-catenin signaling, DNA damage, telomeres, antiviral response, apoptosis, autophagy, glucose metabolism, TNKS inhibitor

## Abstract

Tankyrases (TNKS1 and TNKS2) are multifunctional enzymes of the poly(ADP-ribose) polymerase (PARP) family that regulate cellular homeostasis by catalyzing poly(ADP-ribosyl)ation and stabilizing protein–protein interactions through their ankyrin repeat clusters. By engaging with diverse sets of proteins, TNKSs act as central hubs that coordinate signaling and metabolic pathways. In this review, we discuss how TNKS –protein interactions underpin their roles across multiple biological pathways, including Wnt/β-catenin, YAP and SRC signaling, mTORC1 signaling, DNA damage repair (via PARP crosstalk and recruitment of repair factors), telomere maintenance, cell-cycle regulation, glucose metabolism, cytoskeleton rearrangement, autophagy, proteasomal degradation, and apoptosis. We highlight the structural basis of these interactions, emphasizing ankyrin repeat domain recognition motifs and the consequences of TNKS-mediated PARylation on protein stability and localization. By integrating findings from oncology, virology, and metabolism, we illustrate how TNKS functions as a nodal regulator linking genome stability, signaling fidelity, and metabolic control. The interplay between TNKS and these varied pathways is essential for the well-being of the organism, with its dysregulation having severe biological and clinical consequences, which are discussed here. Finally, we consider therapeutic implications of disrupting TNKS–protein interactions, with particular attention paid to selective small-molecule inhibitors and their translational potential in cancer, viral infections, and degenerative diseases.

## 1. Introduction

TNKS1 and 2 are multidomain members of the poly(ADP-ribose) polymerase PARP super family (PARP5a/b) that post-translationally modify themselves and target proteins with mono- or poly(ADP-ribose) using NAD+ as a substrate (termed MARylation or PARylation, respectively) [1,2]. By recruiting diverse binding partners, TNKS enzymes function as central hubs that coordinate a broad set of cellular pathways, including Wnt/β-catenin signaling, mitotic control, telomere maintenance, and DNA damage repair [3,4,5,6]. Although originally characterised for their roles in telomere regulation and Wnt signalling, recent work has expanded their functional scope to include metabolism, proteostasis, cell fate decisions, and innate immunity [7,8].

TNKS-dependent regulation arises from two interconnected features: selective protein recognition via ankyrin repeat clusters (ARCs), which bind short TNKS-binding motifs (TBMs), and catalysis by the PARP domain, which can modulate substrate stability, localisation, or assembly of macromolecular complexes [9,10,11]. These properties allow TNKS1/2 to act both as enzymes and as structural organisers that determine signaling thresholds across cellular compartments.

The expanding TNKS interactome and the discovery of RNF146-mediated PAR-dependent ubiquitylation (PARdU) have positioned TNKS as key regulators linking proteasomal degradation, genome maintenance, and metabolic homeostasis [3,8,12,13,14]. Their involvement in tumorigenesis, neurodegeneration, viral infection, and metabolic disorders has in turn prompted the development of small-molecule TNKS inhibitors with emerging therapeutic potential [15,16,17,18,19].

**Figure 1:**
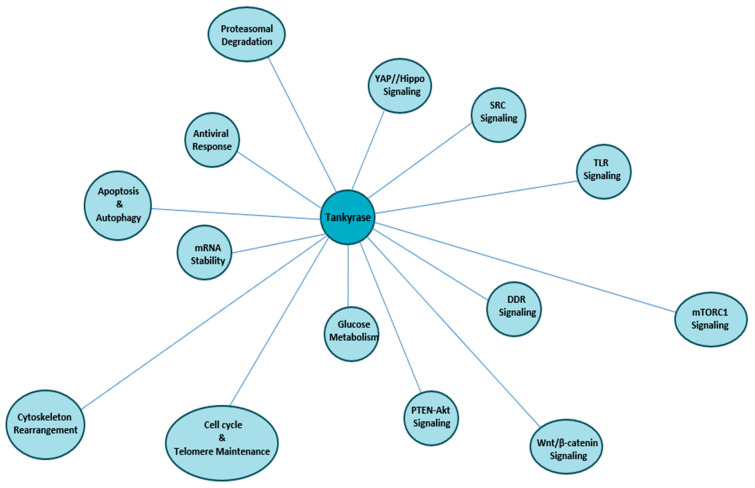
Tankyrase regulation across diverse cellular pathways. The diagram illustrates the multifaceted regulatory functions of TNKS in key cellular processes. Central to numerous signaling and repair networks, TNKS integrates cues from DNA damage repair, telomere maintenance, Wnt/β-catenin signaling, and PTEN–Akt signaling, extending its influence on pathways governing apoptosis, YAP signaling, TLR signaling, mTORC1 signaling, glucose metabolism, autophagy, cytoskeleton rearrangement, and proteasomal degradation. TNKS also modulates the cell cycle, particularly metaphase regulation, and contributes to antiviral responses. Its roles encompass control of protein stability via PARylation and ubiquitylation, modulation of transcriptional and post-transcriptional processes, and coordination of metabolic and proliferative responses to maintain cellular homeostasis.

This review summarises how TNKS–protein interactions underpin these diverse functions and examines structural principles that govern substrate recognition and activation. We then discuss TNK’s roles across major biological pathway homeostasis (as summarized in Graphical Abstract Figure 1), highlight crosstalk between signaling and DNA repair networks, and consider the wider implications for disease and pharmacological targeting.

## 2. Structural Insights into Tankyrase-1 and Tankyrase-2

TNKS1 and TNKS2 are members of the diphtheria toxin-like ADP-ribosyltransferase (ARTD) family of poly(ADP-ribose) polymerases [20,21], critical for telomere maintenance, Wnt/β-catenin signaling, and mitotic progression [22,23]. TNKS1 contains an N-terminal histidine–proline–serine (HPS) domain, absent in TNKS2, which may have a role in protein interactions.

**Figure 2:**
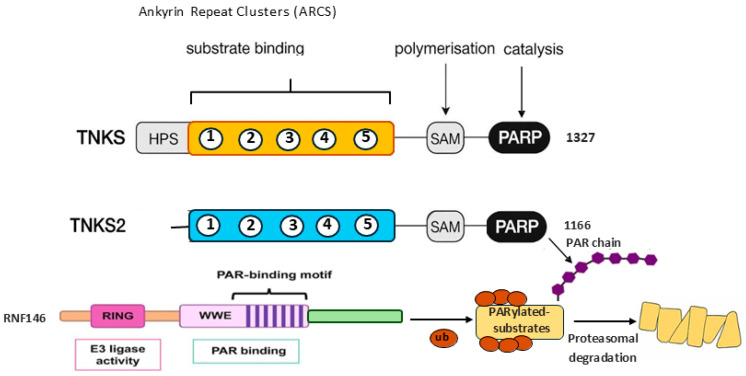
Domain structures of mammalian TNKS1/2 and its functional partner. TNKS recruits its binding partners through ankyrin repeat clusters (ARCs), which recognize degenerate peptide motifs present in numerous proteins. Many of these partners are subsequently PARylated by the PARP domain of TNKS. Previous studies elucidated the mechanism of substrate recognition and demonstrated how mutations in this process underlie the rare human disease Cherubism. The sterile alpha motif (SAM) domain mediates filamentous polymerization of TNKS, a process essential for both its catalytic and non-catalytic functions RNF146 presents two characterized domains in the N-region: the RING domain responsible for the E3-ligase activity and the WWE domain, which recognizes the iso-ADP-ribose moiety present in the PARylated substrates via its PAR-binding motif.

Both proteins share three major functional modules: an ankyrin repeat domain (ARD) with five ankyrin repeat clusters (ARCs 1–5), a sterile alpha motif (SAM) domain, and a C-terminal ADP-ribosyltransferase (ART) domain (Figure 2) [9,24,25]. The ARCs mediate substrate recognition by binding to short peptide motifs in partner proteins. The SAM domain promotes oligomerization and assembly of higher-order protein complexes. The ART or PARP catalytic domain catalyzes the transfer of ADP-ribose units from NAD^+^ onto substrate residues, forming linear or branched poly(ADP-ribose) (PAR) chains. The ARD (~20 repeats for both TNKS1 and TNKS2) clustered into five ankyrin-repeat clusters (ARC1–5) acts as a scaffold [26]. Apart from ARC3, which has no TNKS-binding capability, ARC1,2,4 and 5 contain conserved peptide-binding pockets that recognize TNKS binding motif (TBMs) in substrates like AXIN, following an RXXPDG-like consensus (extended to RXX[A/X] DGX[DE]) [9,27]. Essential arginine and glycine residues in TBMs enable micromolar-affinity interactions via hydrogen bonding and hydrophobic contacts, with enhanced avidity in multivalent TBM substrates [9,25,27]. TBMs can be classified into three categories [9,24,28]. Conventional TBMs (RXXPDG “hexapeptides”) are high-affinity motifs that strongly engage ARC2/4/5 and position substrates for efficient PARylation. Unconventional TBMs deviate from the consensus sequence, leading to weaker or context-dependent ARC binding. Extended TBMs contain flanking residues that enhance affinity and specificity; RNF146/Iduna, which is discussed in detail below, provides a notable example, with its extended TBM optimized for ARC interaction [29,30,31].

The type of TBM profoundly influences both the extent and outcome of TNKS-dependent PARylation of substrates (Supplementary Table S1). Conventional and extended TBMs typically promote robust, often branched PAR synthesis that efficiently recruits the tryptophan-tryptophan-glutamic acid (WWE) domain of RNF146, resulting in ubiquitylation and proteasomal degradation. In contrast, unconventional TBMs usually generate weaker or more transient PARylation, insufficient to engage RNF146, thereby favoring substrate stabilization through interactions with PAR-binding scaffold or repair proteins, particularly in nuclear or mitotic environments [10,11,31,32,33]. Both TNKSs exhibit dynamic subcellular localization that varies across the cell cycle. During interphase, they mainly co-localize with TRF1 at telomeres, with a subset localizing to nuclear pore complexes [34,35]. Upon mitotic entry, coinciding with nuclear envelope breakdown and disassembly of nuclear pore complexes, TNKS1/2 relocate to the pericentriolar matrix of mitotic centrosomes, where they contribute to the regulation of spindle pole assembly and the DNA damage repair pathway [5,34,36,37]. TNKSs are found not only in the nucleus but also in the cytoplasm. The TNKS PARylome extends to multiple cytoplasmic components of the WNT, YAP/Hippo, PTEN–Akt, and SRC/immune signaling pathways, as well as proteins involved in glucose metabolism, and the cytoskeleton. Cytoplasmic localization patterns are also observed at the Golgi apparatus [38]. These distinct subcellular localizations of TNKSs influence the fate of their substrates. For example, cytoplasmic substrates such as AXIN and AMOT undergo degradation to control Wnt/β-catenin and YAP/Hippo signaling, whereas nuclear or mitotic targets, including TAB182 and MDC1 are stabilized to sustain chromosome segregation and DNA repair [22,39]. External stimuli, including oxidative stress, DNA damage, and nutrient deprivation, further modulate TNKS localization, catalytic efficiency, and substrate availability.

Mechanistically, TNKS-dependent PARylation can be viewed as a tunable system that routes substrates either towards proteasomal destruction or towards stabilization, depending on PAR chain density, ubiquitin linkage type, and the local ubiquitin–deubiquitinase balance [40,41]. Upon TBM–ARC engagement, the TNKS ART domain ‘writes’ PAR chains whose length and branching define an avidity threshold for WWE-domain E3 ligases such as RNF146. Once this threshold is reached, RNF146 installs K48-linked ubiquitin chains, committing the substrate to degradation, a process dominant in cytoplasmic hubs such as Wnt and Hippo signaling [10,11]. Conversely, short or sparsely branched PAR often acts as a retention or localization cue, attracting PAR ‘readers’ and scaffolds that promote mono- or K63-linked ubiquitylation and stabilization, as observed in nuclear or mitotic assemblies such as DNA damage foci and spindle poles [42]. PAR-degrading enzymes such as PARG and ARH3 additionally ‘sculpt’ PAR chain length and branching, controlling whether a substrate remains below or above the recognition threshold [43,44,45].

Beyond proteolysis, TNKSs can direct other outcomes: for example, inhibition occurs when substrates such as GDP-mannose 4,6-dehydratase (GMD) bind and suppress TNKS activity [46], whereas PARP-independent scaffolding functions promote higher-order assemblies such as PEX14–ATG9A complexes [47].

Together, these mechanisms position TNKS as context-dependent molecular switches that integrate TBM affinity, ARC selectivity, PAR chain architecture, and subcellular localization to determine whether a substrate is degraded, remodeled, or stabilized.

The ~70-residue, all-α-helical SAM domain mediates head-to-tail polymerization, forming helical assemblies observed at ~3–4 Å resolution by cryo-electron microscopy, that further cluster into cytoplasmic and nuclear puncta or condensates. Polymerized TNKS promotes catalytic activity and substrate scaffolding [12,48].

Beyond serving as a passive scaffold for substrate recruitment, the ankyrin-repeat and SAM domains together define a regulated activation mechanism for TNKSs. Accumulating structural and biochemical evidence indicates that TNKS1/2 activity is not constitutively “on” but is instead triggered through engagement with binding partners that promote higher-order assembly [9,49]. Initial recognition of TBMs by ARC2/4/5 concentrates TNKS molecules on multivalent substrates or scaffolds, such as AXIN, TRF1 dimers, TAB182, or MERIT40-containing repair complexes. This multivalent ARC–TBM engagement increases local TNKS concentration and promotes SAM-domain-mediated head-to-tail polymerization which drives the transition from a closed to an open configuration [12,48,50,51]. Cryo-electron microscopy and mutational analyses have demonstrated that SAM polymerization is a prerequisite for robust catalytic activity, as disruption of SAM–SAM interfaces markedly reduce PARylation despite intact substrate binding [12,48,50,51].

The PARP domain, with a characteristic fold of two anti-parallel β-sheets flanked by α-helices, contains a conserved histidine, tyrosine, glutamic acid (HYE) catalytic triad (e.g., H1031, Y1060, E1138 in TNKS2) that catalyzes NAD^+^-dependent poly(ADP-ribosyl)ation (PARylation), forming predominantly linear PAR chains [52,53,54]. A CHCC-type zinc-binding motif stabilizes the domain. The active site includes a donor site for NAD^+^ (with nicotinamide and adenosine subsites regulated by a flexible D-loop) and an acceptor site for PAR chain elongation. Oligomerization and dimerization of TNKS acts as an allosteric switch for the PARP domain. Higher-order assembly stabilizes the catalytic D-loop and reconfigures the NAD^+^ binding pocket, thereby enhancing both auto-PARylation and substrate-directed PAR chain elongation [18,27,43,44,55,56]. In this model, TNKS-binding proteins function as upstream activators rather than passive substrates: by clustering TNKS molecules and enforcing defined geometries, they license PARP activity only in appropriate spatial and signaling contexts. This explains why isolated TNKS exhibits weak basal activity in vitro, whereas engagement with multivalent TBM-containing partners or forced dimerization markedly enhances catalytic output [12,48,50,51]. Once TNKS is activated, TNKS-driven PARylation can then be further amplified or terminated through auto-PARylation, PAR turnover by hydrolases, and RNF146-mediated ubiquitylation, providing multiple regulatory checkpoints downstream of the initial binding event [11,12,24,57,58].

The linear, negatively charged PAR chains function as dynamic molecular scaffolds, recruiting positively charged effector proteins including those harboring arginine/glycine-rich (RGG) domains, through non-covalent electrostatic interactions [59,60,61,62]. As detailed in the subsequent sections, this charge-based recognition mechanism facilitates the assembly and stabilization of functional complexes in Wnt signaling (via AXIN) [63], mitotic progression (via NuMA and TAB182) [64,65], and DNA damage response (via MDC1 and TAB182) pathways. In parallel, TNKSs form a functional partnership with the E3 ubiquitin ligase RNF146 to trigger polyubiquitylation and proteasomal degradation of the substrate [9,11,63,66,67].

## 3. Regulatory Networks Governing Tankyrase Activity, Stability, and Turnover

TNKS1 level and activity are tightly regulated through a multilayered network that coordinates its activation, modification, and degradation to ensure precise control of its cellular functions. A key self-regulatory mechanism involves auto-PARylation, through which TNKS1 catalyzes the addition of poly(ADP-ribose) polymers onto itself. This modification modulates the enzyme’s catalytic activity, structural conformation, and turnover. While transient auto-PARylation enhances TNKS1 activity and substrate binding, extensive PARylation can serve as a degradation signal by recruiting PAR-binding E3 ubiquitin ligases [12,19,39]. Upstream kinase-mediated regulation also fine-tunes TNKS1 function. Polo-like kinase-1 (Plk1) phosphorylates TNKS1 to enhance its stability and localization to centrosomes and telomeres during mitosis. Glycogen synthase kinase-3 (GSK3) and mitogen-activated protein kinase (MAPK) contribute additional phosphorylation events that modulate TNKS1’s interactions and catalytic output in response to growth and stress signaling [6,23,68]. A new study demonstrates that phosphorylation within the TBM, particularly at serine 8, markedly enhances affinity for TNKS ankyrin repeat clusters, suggesting a regulatory layer for effector recruitment. Phospho-enrichment analyses indicate that this modification may operate in a context-specific manner, especially at centrosomal complexes. These findings support a model in which TBM phosphorylation tunes TNKS engagement and complex stability [69]. TNKS turnover is primarily governed by the ubiquitin–proteasome system. Following auto-PARylation, RNF146 mediates Lys48-linked ubiquitylation, targeting TNKS1 for degradation by the 26S proteasome. Other E3 ligases, including Deltex (DTX), RNF114, RNF166, and RNF8, also regulate TNKS1 ubiquitylation in different cellular contexts, collectively shaping its half-life and subcellular distribution [3,63,70]. Conversely, the deubiquitylase (DUB) USP25 counteracts these effects by removing degradative ubiquitin chains from TNKS1, thereby maintaining its stability and PARylation potential [71].

Together, these interconnected phosphorylation, PARylation, ubiquitylation, and deubiquitylation events constitute a dynamic regulatory network that balances TNKS1 activation and turnover, ensuring its precise role in, for example, telomere maintenance, Wnt-signaling, and mitotic progression.

## 4. Tankyrase and the Wnt Signaling Pathway

The Wnt signaling pathway is a conserved cascade critical for development and tissue homeostasis broadly divided into canonical (β-catenin-dependent) and non-canonical (β-catenin-independent) branches [72,73,74]. Non-canonical pathways, such as the planar cell polarity (PCP) pathway (regulating cytoskeletal dynamics via Rho GTPases) and the Wnt/Ca^2+^ pathway (modulating intracellular calcium for migration and polarity), operate independently of β-catenin, often antagonizing canonical signaling. Non-canonical Wnt pathways, particularly those activated by Wnt5a, have emerged as key regulators of inflammatory signaling and programmed cell death, with the MAP3K7 kinase TAK1 acting as a pivotal mediator of these responses [73,75,76]. TAK1 predominantly modulates non-canonical Wnt signaling, whereas TNKS amplifies canonical Wnt/β-catenin activity by PARylating AXIN and promoting its RNF146-mediated degradation [3,63]. Together, these enzymes coordinate distinct branches of the Wnt network and shape inflammatory output, cell survival, and tissue homeostasis.

**Figure 3:**
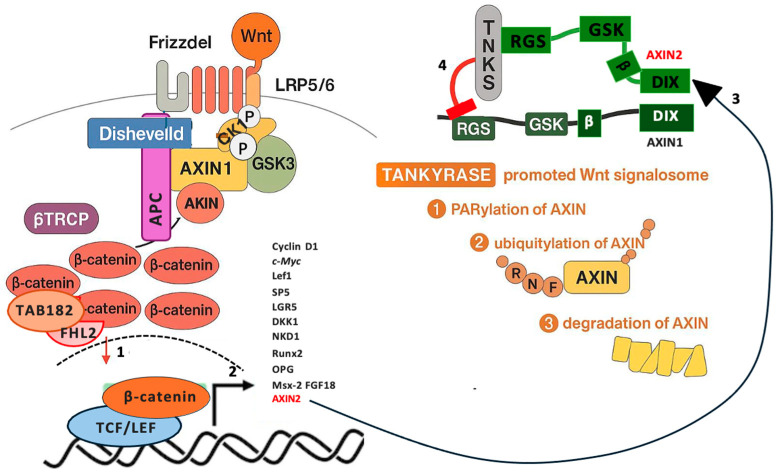
Tankyrase-mediated regulation of Wnt/β-catenin signaling pathway. Upon Wnt stimulation, Wnt ligands engage Frizzled receptors together with LRP5/6 co-receptors, leading to activation of Dishevelled (DVL). AXIN binds to phosphorylated LRP within Wnt signalosomes, signaling for PARylation by TNKS and inhibition of the β-catenin destruction complex. This permits cytoplasmic stabilisation and accumulation of β-catenin. **(1)** The TAB182–FHL2 complex subsequently associates with β-catenin, promoting its translocation into the nucleus and enhancing activation of TCF/LEF-dependent transcriptional programmes. **(2)** Co-activation of TCF/LEF drives the expression of proliferation- and stemness-associated genes, including c-Myc, Cyclin D1 and AXIN2. **(3) & (4)** In turn, AXIN2 recruits tankyrase to AXIN1-containing condensates, facilitating AXIN1-specific PARylation and turnover, thereby limiting AXIN1 stability. PARylated AXIN1 is recognised by the E3 ubiquitin ligase RNF146 and subsequently targeted for proteasomal degradation. The solid bold line represents the cell membrane, while the dashed line indicates the nuclear membrane.

In the canonical pathway, Wnt ligands bind Frizzled receptors and LRP5/6 co-receptors to activate Disheveled (DVL), inhibiting the β-catenin destruction complex and allowing β-catenin accumulation for nuclear translocation and co-activation of TCF/LEF transcription factors; this promotes expression of genes involved in proliferation and stemness, such as *c-Myc* and *Cyclin D1* (Figure 3) [77,78,79]. Mutations in the *CTNNB1* gene, which encodes β-catenin, are reported in about 20–30% of hepatocellular carcinoma (HCC) cases as a direct indicator of Wnt pathway disruption [80].

The β-catenin destruction complex, or degradasome, is a multiprotein assembly stabilized by a scaffold of AXIN1/AXIN2 and APC, with GSK3α/β and CK1 phosphorylating β-catenin. This facilitates ubiquitylation by β-TrCP and proteasomal degradation in the absence of Wnt [81,82]. APC enhances AXIN multimerization and GSK3 recruitment, while truncation of APC (occurring in >80% of colorectal cancers (CRC) disrupts this, leading to β-catenin hyperactivation [81,82,83,84]. TNKS1/TNKS2 regulate canonical Wnt/β-catenin signaling by PARylating key degradasome components (AXIN1/2 and APC), primarily through conventional TBMs that bind ARCs (ARC2/4/5), resulting in functionally regulated PARylation patterns that drive degradation or modulation. AXIN1 and AXIN2, core scaffold components, undergo robust PARylation with long/branched chains marking them for RNF146-mediated ubiquitylation and degradation, thus, destabilizing the complex, which, in turn, stabilizes β-catenin upon Wnt stimulation [3,9,23,66,85].

TNKS1 Binding protein 1 182 KDa (TNKS1BP1 or TAB182) inhibits the phosphorylation of β-catenin by GSK3β and interacts with four-and-a-half LIM-only protein 2 (FHL2), which in turn promotes the nuclear translocation of β-catenin and activates the transcription of downstream TCF/LEF-dependent target genes [86,87]. TAB182 protein includes a nuclear localization signal (NLS) which helps with localization in both the nucleus and cytoplasm. To date, no study has demonstrated that TAB182 associates with TNKS specifically during β-catenin translocation to the nucleus, and such a regulatory role remains unproven. TAB182 and FHL2 are interchangeable cofactors associated with the CCR4-NOT complex, a multifunctional regulator of mRNA metabolism and gene expression [88,89]. The core subunits of the CCR4-NOT complex (CNOT2 and CNOT3) have been shown to act as positive modulators of Wnt/β-catenin signaling pathway [90].

In the assembled degradasome, AXIN phosphorylation limits its accessibility to TNKS, while upon Wnt stimulation this phospho-protection is lifted by inhibiting CK1/GSK3 activity and phosphorus removal by protein phosphatase 1 (PP1) [91,92,93]. In mammals, AXIN2 functions as the principal scaffold, drawing TNKS into AXIN1 condensates to enable AXIN1-specific PARylation and turnover, thereby shaping overall Wnt signaling output. Whereas AXIN1 overexpression does not lower β-catenin levels, AXIN2 overexpression can promote invasive behavior, reflecting AXIN2’s context-dependent tumor-suppressive and oncogenic properties. When both AXIN proteins are lost, degradasome assembly collapses, leading to increased β-catenin accumulation [94,95,96,97,98]. APC, with a speculative conventional TBM, promotes AXIN multimerization but is not globally degraded in response to TNKS PARylation (unlike *Drosophila* APC2), though TNKS inhibitors (TNKSi) restore AXIN stability and multimerization in APC-mutant CRC, showing promise as anti-cancer drugs [19,99]. TNKS’s dual PARylation of AXIN2, stabilizing it upon Wnt activation by boosting AXIN1 degradation yet degrading it in the cytoplasmic degradasome, exemplifies its compartmentalized regulation, where TNKS inhibition globally increases AXIN levels (but not APC), underscoring its therapeutic potential in Wnt-driven cancers.

TNKSs directly interact with NKD1 and NKD2, promoting their degradation [22]. NKD1 and NKD2 are intracellular inhibitors of the canonical Wnt/β-catenin signaling pathway; they bind to DVL to prevent β-catenin from accumulating in the nucleus and activating target gene transcription. The loss or degradation of NKD1 and NKD2 removes this inhibitory control, thereby enabling activation of the Wnt/β-catenin pathway [100,101].

## 5. Tankyrase and DNA Damage Repair (DDR) Pathways

Genomic DNA is continually challenged by endogenous and exogenous insults, including reactive oxygen species, chemicals, and ionizing radiation, producing single-strand breaks (SSBs), double-strand breaks (DSBs), and base damage. If unrepaired, these lesions compromise genome integrity and cell viability. Eukaryotic cells therefore deploy a coordinated DNA damage response (DDR) as a hierarchical and highly coordinated process in which early damage sensors initiate signaling cascades that recruit downstream repair, checkpoint, and chromatin-remodeling factors. Members of the PARP family are central to this response; however, TNKS1/2 operate through a fundamentally different mode of action from canonical DNA damage sensors such as PARP1. [102,103]. PARPs are key coordinators of the DNA damage repair response, rapidly detecting DNA strand breaks and generating poly(ADP-ribose) chains that recruit and organize repair factors at damaged sites. Through this PARylation-driven signaling, PARPs facilitate chromatin remodeling, stabilize repair complexes, and ensure timely progression of both single- and double-strand break repair pathways [104,105]. Unlike PARP1 and PARP2, which directly recognize broken DNA ends and are catalytically activated by DNA binding, TNKS1/2 are not primary DNA-binding proteins in the context of DSBs [9,14]. Instead, TNKS1/2 are recruited indirectly to sites of DNA damage through protein–protein interactions within pre-assembled or damage-induced repair complexes. This recruitment is mediated by the ankyrin repeat clusters (ARCs) of TNKS1/2, which recognize defined TNKS1/2 -binding motifs or motif-like sequences present on DDR-associated proteins [27]. Through ARC-dependent binding, TNKS1/2 are incorporated into repair assemblies only after early DNA damage signaling has been established, positioning them downstream of lesion recognition. Rather than functioning redundantly, these interactions place TNKSs within distinct repair sub-complexes, allowing them to influence both homologous recombination (HR) and NHEJ in a context-dependent manner. Once recruited, TNKS1/2 regulate DNA repair primarily by modulating the stability, organization, and spatial dynamics of repair complexes rather than by acting as catalytic initiators [5,27]. In nuclear and mitotic contexts, TNKS-dependent PARylation typically promotes assembly and stabilization of repair complexes (damage foci) rather than RNF146-driven turnover [106,107].

Key adaptors and scaffold proteins act as molecular bridges that recruit TNKS to damaged chromatin. MDC1, which accumulates on γH2AX-marked chromatin following ATM activation, provides a central platform for DDR assembly and mediates TNKS recruitment to DSB sites [5].

**Figure 4:**
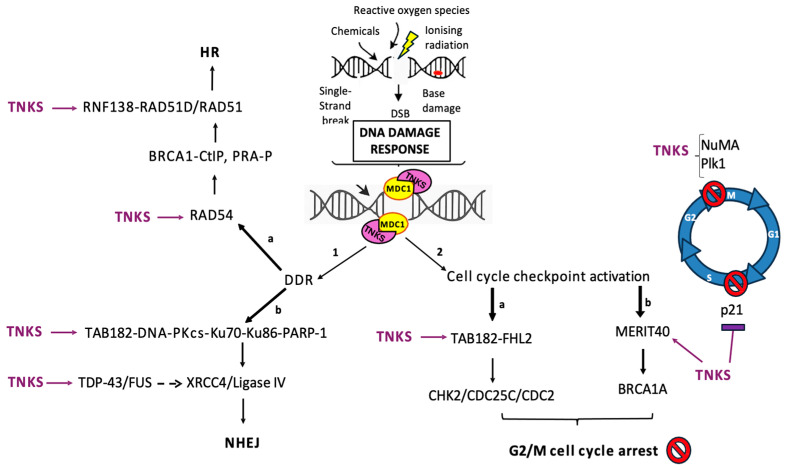
Tankyrase-mediated regulation of DNA damage Repair signaling pathway. When DNA damage occurs, cells activate a coordinated response to repair the lesions and preserve genomic integrity. TNKSs are integral components of the broader DDR network, influencing the balance between non-homologous end joining (NHEJ) and homologous recombination (HR) repair pathways. MDC1 recruits TNKS to double-strand break (DSB) sites. TNKS2 also interacts with RAD54 and may function in a regulatory or scaffolding capacity to facilitate the recruitment of additional DNA damage response components. **(1.a)** The MDC1–TNKS interaction stabilizes the BRCA1A protein complex, comprising BRCA1, RAP80, and MERIT40 at sites of DNA damage. Proper localization of this complex is essential for efficient HR, which relies on the generation of single-stranded DNA ends required for repair. Depletion of TNKS impairs the recruitment of resection-associated proteins such as CtIP. **(1.b)** Regulation of NHEJ by TNKS occurs indirectly through TAB182 and RNA-binding proteins (RBPs) TDP-43 and FUS. TAB182 interacts directly with DNA-PKcs and the Ku70/Ku86 heterodimer, which recognises DSBs. The TAB182–PARP1 interaction enhances kinase activity of DNA-PKcs, while the TAB182–TNKS interaction modulates DNA-PKcs stability and promotes its activation. Additionally, the RBP TDP-43 functions as a scaffold to recruit the XRCC4/Ligase IV complex to damage sites for end ligation. TNKSs also influence cell cycle progression at the G1/S and G2/M phases. Elevated TNKS expression promotes RNF146-dependent ubiquitylation and proteasomal degradation of p21, facilitating the G1/S transition. **(2.a)** TAB182 interacts with FHL2 to regulate the G2–M checkpoint through wiring the CHK2/CDC25C/CDC2 signaling pathway. **(2.b)** In parallel, TNKSs cooperate with RNF8 to stabilize the BRCA1A complex via MERIT40 at DSBs, contributing to activation of the G2/M checkpoint. During mitosis, TNKS1 regulates spindle assembly and chromosome segregation through PARylation of Plk1 and NuMA proteins, ensuring accurate mitotic progression. Solid arrows indicate direct interactions, whereas dashed arrows represent indirect interactions.

TNKS1/2 facilitate the recruitment of RAD51 to DNA DSBs, promoting HR (Figure 4). Depletion of TNKSs impairs RAD51 loading, resulting in reduced HR efficiency comparable to that observed with BRCA1 depletion [5]. TNKS1/2 recruit the CtIP–BRCA1 complex to chromatin, where CtIP mediates DNA end resection. This has been shown to be dependent on MDC1, as the TNKS1-binding site (amino acids 948–955 and 1993–2000 in the central and C-terminal regions) mutated form of MDC1 fails to recruit BRCA1, in contrast to the wild-type protein [5]. The BRCA1 complex contains MERIT40 and RAP80 [5]. TNKS1/2 act in parallel with RNF8 to stabilize the BRCA1 complex via MERIT40 at DSBs and activate the G2/M checkpoint. Interestingly, both wild-type and catalytically inactive (PARP-dead) forms of TNKSs restore HR efficiency, indicating a structural rather than enzymatic role for TNKSs in HR-mediated DNA repair [5]. Consistent with this, inhibition of TNKS PARP activity using XAV-939 has no detectable effect on HR. PARylation of RAD54, a Swi2/Snf2-family ATP-dependent DNA translocase, is essential for HR, in that it is involved in remodeling of chromatin, and promotion of RAD51 filament function. TNKS2 also interacts with RAD54 but is not detectably PARylated suggesting that their association may serve a regulatory or scaffolding rather than a catalytic role [5]. RNF138 is an E3 ubiquitin ligase that facilitates homologous recombination (HR)-mediated repair of DNA double-strand breaks. It is recruited to DNA damage sites through its C2H2-type zinc finger domains, where it promotes HR by stimulating DNA end resection [108,109]. Mechanistically, RNF138 mediates the displacement of the Ku heterodimer, which otherwise obstructs resection and enhances the stability and activity of CtIP, a key resection factor [108,109]. Furthermore, RNF138 interacts with, and ubiquitylates, RAD51D, a component of the RAD51 paralogue complex, thereby further supporting the recombination process. Following treatment with TNKS inhibitors, RNF138 was observed to co-immunoprecipitated with reduced levels of TNKS1 [41]. The absence of detectable alterations in TNKS1 abundance or post-translational modification may reflect the relatively low endogenous expression of these proteins, as well as the dependence of their association on TNKS1’s catalytic activity [110,111].

TAB182 links TNKS to DNA-PKcs-centered assemblies involved in NHEJ and functions as a scaffold that promotes mitotic and repair assemblies in a TBM-dependent manner. Emerging research suggests TNKS-TAB182 interaction may cause a feedback loop where the proteins influence each other’s activity and stability [112,113]. 

Several studies have shown that TAB182 is involved in the repair of DNA double-strand breaks (DSBs) and may serve as a potential therapeutic target to enhance the radio- and chemosensitivity of various tumors. For example, TAB182 regulates irradiation-induced phosphorylation of DNA-PKcs and facilitates DSB repair by modulating the interaction between PARP-1 and DNA-PKcs [37]. TAB182 enhances the radio-resistance of esophageal squamous cell carcinoma (ESCC) cells by interacting with FHL2 to regulate the G2–M checkpoint through wiring the CHK2/CDC25C/CDC2 signaling pathway [114]. TAB182 was reported to be upregulated in human lung adenocarcinoma (LAC) tissues, and this was associated with poor outcomes in LAC patients due to dysregulation of HR of DSBs [115]. As mentioned earlier, TAB182 represents one of the proteins that functionally link the DDR pathway with Wnt signaling.

Additional components highlight how TNKS-centered PAR signaling interfaces with genome maintenance at multiple levels. The transactive response DNA binding protein of 43KDa (TDP-43) and Fused in Sarcoma (FUS), are DNA- and RNA-binding proteins (RBPs) involved in transcriptional regulation, RNA metabolism, and protein translation [116,117]. They both include an RNA recognition motif (RRM), which facilitates binding of both FUS and TDP-43 to PARPs, including TNKSs, although by different mechanisms. FUS has been shown to have no specific TNKS-binding domain and presumably non-covalently interacts with TNKS through a combination of PAR and RRM/motif binding. In this context, the transient interactions between PAR and FUS may prime FUS to drive homotypic FUS–FUS multimerization [118]. This interaction is also essential for FUS to be recruited to sites of DNA damage and to form protein condensates, but the exact mechanisms and specific motifs involved are still being investigated. Unlike FUS, the TNKS binding site comprises residues 165–170 (RHMIDG), present in RRM1 of TDP-43, with the histidine and isoleucine amino acids being of central importance to the interaction [119] . Previous findings indicated that it is not targeted for degradation via TNKS1/2-dependent ubiquitylation, as discussed in more detail below [17,120]. Similar to FUS, TDP-43 interaction with TNKS results in its stabilization and is a key step in its recruitment to sites of DNA damage and its role in forming biomolecular condensates which probably contribute to the formation of DNA repair foci [121,122,123,124,125,126]. TDP-43 is swiftly recruited to DSB sites alongside early DDR factors, including phospho-ATM and γH2AX, as well as core NHEJ proteins such as the Ku heterodimer. In healthy neurons, TDP-43 is essential for facilitating the rate-limiting final step of DSB repair by promoting the recruitment of the XRCC4/Ligase IV complex to the damage sites [121]. Both FUS and TDP-43 undergo liquid–liquid phase separation (LLPS) to form biomolecular condensates, such as stress granules, in both healthy and diseased states. However, their condensation is governed by distinct molecular grammars. Inhibition of TNKS leads to a reduction in the formation of cytoplasmic TDP-43 foci, although stress granule formation is unaffected. It has been suggested that as inhibition of TNKS with small molecules mitigates against TDP-43-associated neurodegeneration, their clinical use against Amyotrophic Lateral Sclerosis (ALS) (also known as Motor Neuron Disease (MND) and FTD may be possible [17,127,128].

PARP1-dependent PARylation of DNA-PKcs, a key enzyme in NHEJ, has been associated with enhanced kinase activity, whereas TNKSs have also been found to regulate DNA-PKcs stability and promote its activation. The relationship between them appears to involve TNKS-mediated control of DNA-PKcs activity and protein levels, underscoring their interconnected roles in DNA repair and the cellular response to DNA damage [36].

The cell cycle regulator p21 interacts with TNKS and is PARylated both in vivo and in vitro [129]. Two TNKS binding motifs have been identified on p21: RQNPCG and RVRGLG sequences located at residues 9–14 and 67–72, respectively, although RQNPCG appears to be more important for the association [129]. Increased expression of TNKSs results in ubiquitylation of p21 through RNF146 and subsequent proteasomal-mediated degradation. Thus, inhibition of TNKS has been suggested to stabilize p21 in the G1 phase of the cycle, allowing continued E2F/Rb complex stability, downregulation of cell cycle genes and cell cycle arrest [129]. At the mRNA level, reduction in two CCR4–NOT subunits, CNOT1 (the scaffold subunit) and CNOT6L (deadenylase subunit), leads to G1 cell cycle arrest through increased stability of the G1 cell cycle inhibitors p21/Cip1 and p27/Kip1, respectively [130,131]. Whether TNKS-dependent PARylation via TAB182 signals for the recruitment of the CCR4–NOT complex to 3′UTR mRNA targets has not yet been investigated. However, PARP1-dependent PARylation has been shown to influence mRNA target stability and decay [132].TNKS1 is post-translationally regulated by Polo-like kinase-1 (Plk1), a central mitotic kinase governing spindle organization and chromosome segregation. Phosphorylation of TNKS1 by Plk1 on multiple serine and threonine residues enhances its stability and catalytic activity, preventing proteasomal turnover and promoting localization to centrosomes and spindle poles [6]. There, TNKS1 sustains bipolar spindle assembly by PARylating NuMA. The nuclear mitotic apparatus protein (NuMA) is a key structural component that organizes spindle poles and ensures faithful chromosome segregation during mitosis [133]. Its localization and function are tightly controlled through cell-cycle-dependent post-translational modifications, most notably TNKS-dependent poly(ADP-ribosyl)ation (PARylation) [64,65,112]. TNKS1 associates with NuMA at centrosomes and spindle poles, where PARylation modulates NuMA’s dynamic tethering to microtubules and stabilizes spindle architecture. Inhibition or loss of TNKS activity disrupts NuMA localization, leading to spindle defects, chromosome mis-segregation, mitotic arrest, and micronuclei formation that triggers DNA damage signaling and compromises genome integrity [65,134]. Although NuMA is not a canonical DNA repair factor, its TNKS-mediated regulation integrates mitotic control with the DNA damage response, ensuring that accurate spindle assembly and chromosome segregation are coupled to the maintenance of genomic stability across successive cell cycles [135,136]. Loss of Plk1-dependent phosphorylation destabilizes TNKS1 and results in spindle defects, chromosome misalignment, and delayed mitotic progression. Inhibition of Plk1 further impairs TNKS1 recruitment to telomeres, causing persistent sister-telomere cohesion and metaphase chromosome bridging phenotypes, indicating mitotic stress and genomic instability. The Plk1–TNKS1 axis therefore integrates phosphorylation and PARylation to coordinate spindle dynamics with chromosome segregation fidelity [6,137].

## 6. Tankyrases, Viral Infection and Antiviral Response

The relationship between viruses and TNKSs is complex. TNKSs form part of the host cell antiviral response, PARylating VISA, yet are required to be upregulated during infection by some viruses. Unfortunately, evidence of the relationship is lacking for many virus serotypes, although data has been presented for a limited number of both DNA and RNA viruses. Epstein–Barr virus (EBV) is a double stranded DNA virus, (also known as human herpesvirus 4 (HHV-4), which establishes a lifelong latent infection and has been linked to several cancers, including Burkitt lymphoma and nasopharyngeal carcinoma [138,139]. It was originally shown that TNKS was recruited to the OriP dyad symmetry (DS) region of the EBV genome, regulating episomal maintenance [140,141]. Subsequent studies showed that EBNA1 contained two TBMs located towards its N-terminus and these mediated binding with the ankyrin repeat domain of TNKS [140,141]. Abrogation of interaction between EBNA1 and TNKS enhanced OriP-dependent DNA replication, whereas association between TNKS and EBNA1 inhibits OriP replication in a PARP-dependent manner. In addition, EBNA1 is subject to PARylation in vivo and in vitro [140,141]. In contrast herpes simplex virus-1 (HSV-1) [142], also a double stranded DNA virus, requires TNKS1 for optimal viral replication [143]. During HSV-1 infection TNKS1 is recruited to viral replication centers and co-localizes with ICP0, a multifunctional protein that is responsible for determining the balance between productive lytic replication and reactivation from latency. It has also been shown that there is a direct interaction between the two proteins although the binding sites have not been identified [143].

As well as DNA viruses, certain RNA viruses have a close relationship with TNKSs. TNKS1 and 2 expression are upregulated during infection by Influenza A virus (IAV) [144,145]. Concomitantly, depletion of TNKSs represses IAV replication. In these studies, it was shown that miR-206 is a miRNA that targets TNKS2 and inhibits IAV replication in vitro using various IAV clinical strains such as H1N1 and H3N2. In addition, miR-206 limits viral replication and inhibits expression of viral NP and NS1 proteins in mice [144]. Furthermore, miR-206 inhibits IAV infection in mice by activating an anti-viral state, such that JNK/c-Jun signaling is activated; there was also an increased Stat signaling and an increase in expression of type I and type III mRNAs in these studies. In a complementary investigation, it was observed that miR-9-1 targets TNKS2 and that its overexpression limits IAV replication in vitro [145]. miR-9-1 also activates Stat signaling and induces IFN I expression, favoring an antiviral environment [145]. However, the precise relationship between IAV and TNKSs in this context remains unclear and must await further investigation. In another study of innate immunity, a direct interaction between VISA (virus-induced signal adaptor; also known as mitochondrial antiviral signaling protein, MAVS) and TNKS1 has been observed [16]. TNKS PARylates VISA, attenuating the innate immune response to RNA viruses. Following infection with the single strand RNA virus, Sendai virus (SeV, murine respirovirus, formerly also known as murine parainfluenza virus type 1), TNKS1 and 2 are upregulated and translocated to the mitochondria where they bind to VISA and catalyze PARylation at residue E137 [16]. This facilitates ubiquitylation by RNF146 and proteasome mediated degradation. Reduction in level of TNKSs and/or RNF146 potentiates Sendai virus-induced transcription of downstream antiviral genes, including IFNB1, ISG56, and IL6, compared with controls [16]. A TNKS binding site has been identified on VISA at amino acids 171–175 and this has been shown to interact with the ANK region of TNKS [16].

## 7. Tankyrases and Apoptosis and Autophagy

Apoptosis, a form of programmed cell death, is a universal process in multicellular organisms and is central to their growth and development. TNKSs have been shown to impact apoptosis in a number of studies. However, there is an inconsistency between some reports; for example, overexpression of TNKS has been suggested to either cause cell death in human fibroblasts and tumor cells [146] or to reduce the rate of apoptosis [147]. In the latter, as well as in other studies, it was seen that ablation of TNKS expression, in human tumor cells, promotes apoptosis, reducing proliferation [147,148]. An appreciable number of investigations have indicated that inhibition of TNKSs with specific drugs affects the apoptotic response in human tumor cells and this is generally seen as an increase in apoptosis [149,150,151,152].

TNKS1 binds directly to both the long and short forms of Mcl-1, but not to other members of the Bcl-2 family of apoptosis regulatory proteins [153] Mcl-1L, like Bcl-2 itself, is an anti-apoptosis component of the pathway whereas the shorter form of Mcl-1 (Mcl-1S) is pro-apoptotic [154]. Although Mcl-1 is not a substrate for TNKS-mediated ADP-ribosylation, over-expression of TNKS reduces expression of both forms of Mcl-1 and inhibits both their pro-survival and pro-apoptotic activities [153]. In complementary experiments, it was shown that ablation of TNKS expression in A549 cells led to increased Mcl-1 level and, interestingly, reduced tumor growth when introduced into nude mice [139,147]. In mapping studies, the binding site for TNKS was found to be in the N-terminal region of Mcl-1 and for Mcl-1 the first ten ankyrin domains on TNKS [153]. In complementary studies, using the *Drosophila* model system it was shown that ectopic expression of *Drosophila* TNKS (DTNKS) results in severe and widespread developmental problems and that over-expression of DTNKS in imaginal discs results in caspase dependent apoptosis [155]. In addition, a high level of DTNKS is associated with aberrant expression of apoptosis-regulatory proteins favoring apoptosis; specifically, DIAP1 (Death-associated inhibitor of apoptosis 1) is down-regulated whereas the hid protein (a member of the RHG family which bind and neutralize IAPs [inhibitors of apoptosis]) is upregulated [155,156]. DTNKS also activates JNK signaling which is required for apoptosis [155].

Macroautophagy (commonly shortened to autophagy) is an evolutionarily conserved cellular pathway that controls protein and organelle degradation, and has essential roles in survival, development and homeostasis. Multiple forms of autophagy have been distinguished specific to different organelles, including mitophagy, pexophagy, reticulophagy, ribophagy, lysophagy, and nucleophagy reviewed by [157]. Pexophagy is important for the quality control of peroxisomes and involves their degradation through the autophagy pathway [158]. Several pathways appear to regulate this process, one of which involves the interaction of TNKS with PEX14 to form a ATG9A-TNKS1/TNKS2-PEX14 complex that is involved in a noncanonical pexophagy pathway [47]. Both TNKS1 and 2 bind to PEX14 and co-immunoprecipitate with PEX5. Both TNKSs localize to peroxisomes and promote pexophagy, although there appears to be no necessity for TNKS enzyme activity [47]. Furthermore, TNKS1 and 2 associate and co-localize with the autophagy ATG protein ATG9A to form an ATG9A-TNKS1/2-PEX14 axis which bridges the peroxisome and autophagy machinery to induce pexophagy. Deletion of the TNKS ankyrin domain ablates its ability to localize to peroxisomes and to promote pexophagy and it is presumed that this forms the binding site for PEX14 [157,159]. To what extent TNKSs impact other forms of autophagy is not clear and remains to be determined.

## 8. Tankyrase and Telomere Maintenance

**Figure 5:**
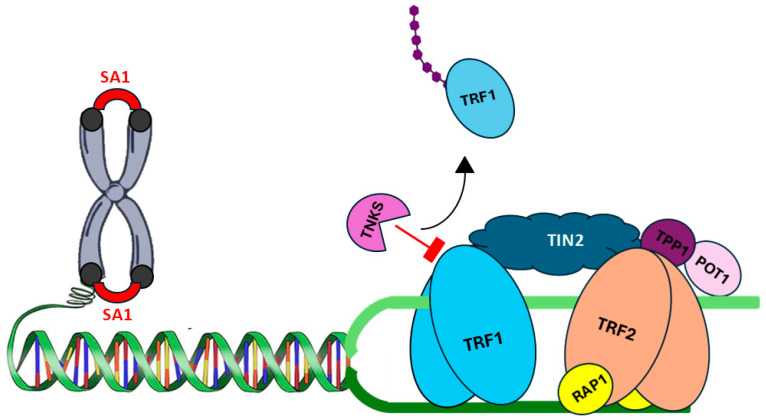
Tankyrase-mediated regulation of Telomere Maintenance. TNKS-mediated regulation of telomere maintenance. TNKS1/2 safeguard telomere homeostasis by coordinating telomerase access and sister-telomere resolution during late S and G2/M. TNKS (particularly TNKS1) promotes the removal of the telomeric shelterin component TRF1 and its associated factors, including the cohesin subunit SA1, at telomeres during mitosis. Through PARylation of TRF1, TNKS1 facilitates the dissociation of the TRF1–TIN2–SA1 complex, which allows sister telomeres to separate properly in mitosis. In essence, TNKS1’s enzymatic activity indirectly regulates the telomere-specific cohesion function of SA1 by modulating its association via the TRF1–TIN2 complex.

TNKS1 and TNKS2 are central regulators of telomere maintenance, coordinating telomerase access, resolution of sister-telomere cohesion, and cell-cycle-linked genome stability [6,160]. Telomeres comprise TTAGGG repeat tracts assembled into the shelterin complex (TRF1, TRF2, TIN2, TPP1, POT1 and RAP1), which shields the telomere 3′ overhang from DNA damage surveillance and restricts access to telomerase, modulating the balance between telomere capping and elongation (Figure 5) [23,161]. Within this network, TRF1 functions as a negative regulator of telomere extension by restricting telomerase access to chromosome ends; significantly, its dissociation from telomeric DNA is tightly controlled by poly(ADP-ribosyl)ation. TNKS-mediated PARylation of TRF1 disrupts its DNA binding, promoting its release and subsequent RNF146-dependent ubiquitylation and degradation, thereby relieving inhibition and enabling telomerase engagement at the 3′ overhang [23,35,162,163]. Increased telomerase activity maintains telomere length and enables cells to bypass replicative senescence (the Hayflick limit) and achieve unlimited proliferative capacity. In cancer, this supports tumour growth, tolerance of genomic instability, and clonal evolution, thereby promoting progression, metastasis, and therapeutic resistance [164].

PARP1-mediated PARylation provides an additional regulatory layer, transiently reducing TRF1 affinity for telomeric DNA and simultaneously creating a docking interface for replication-associated helicases such as WRN and BLM, which resolve secondary DNA structures during S phase. This reversible modification is rapidly removed by PAR-hydrolases, ensuring dynamic turnover of TRF1 at telomeres. Once telomerase is recruited, the TPP1–POT1 subcomplex anchors the enzyme and modulates repeat-addition processivity [165]. Meanwhile, TRF2 preserves telomeric architecture and suppresses inappropriate DNA damage signaling, with PARP1/2 acting primarily in telomeric repair rather than elongation. Together, these mechanisms integrate PARylation-driven shelterin remodeling with controlled telomerase action to promote faithful telomere synthesis and maintain genome stability [166,167]. Beyond its enzymatic role in TRF1 turnover and cohesion resolution, TNKS also operates as a dynamic scaffold capable of assembling multiprotein complexes. Structural studies have shown that although TRF1 contains only a single TBM within each monomer (residues 13–20), its obligate dimeric architecture allows two TBMs to be presented simultaneously, enabling engagement with distinct ankyrin-repeat clusters on TNKS. By contrast, insulin-responsive aminopeptidase (IRAP) and TAB182 harbor multiple partially overlapping TBMs within a single polypeptide, providing intrinsic multivalency that permits binding to different ARC domains on the same TNKS molecule [112,113].

TIN2 acts as a specific PARP modulator within the telomeric protein complex, preventing the premature inactivation and removal of TRF1 by TNKS1, which helps maintain appropriate telomere length homeostasis [168].

TNKS activity peaks in late S and G2/M phases, when cohesion resolution is required; the PARylation of TRF1 causes the TRF1-TIN2-SA1 complex to dissociate. This ensures timely separation of sister telomeres before anaphase [169,170]. SA1 (STAG1) is a telomere-associated cohesion subunit. TNKS1’s mitotic role is reinforced by post-translational mechanisms that increase its stability and telomeric PARP activity, such as Plk1-mediated phosphorylation and RNF8-driven K63-linked ubiquitylation of TNKS1, while the BRISC deubiquitylase complex counteracts the latter to prevent premature cohesion release [6,137]. In parallel, as discussed earlier, TNKS1 also PARylates the spindle-associated protein NuMA. The combined failure of cohesin release and compromised spindle mechanics results in the formation of anaphase bridges between unresolved sister telomeres, which frequently undergo breakage, chromosome mis-segregation, and breakage–fusion–bridge cycles [161,171].

The multivalent nature of TNKSs enables them to bind and recruit multiple substrates simultaneously, forming large molecular assemblies that coordinate telomere maintenance with broader cellular functions such as membrane trafficking, metabolic signaling, and mitotic control. Acting as both enzymes and scaffolds, TNKSs serve as molecular organizers that couple PARylation activity with the spatial regulation of signaling and structural pathways [4,9,36,38,107,171,172].

TNKSs also contribute to telomere maintenance by promoting the repair of damaged telomeric DNA, thereby ensuring robust chromosome end protection and preserving genomic stability. C19orf43, also known as TRIR (Telomerase RNA Component Interacting RNase), is an exoribonuclease that participates in the 3′ end processing of telomerase RNA. It has been observed to associate with TNKS at sites of transcription–replication interference, although the mechanism underlying its recruitment, particularly whether it involves a defined TBM has not yet been established. Emerging evidence indicates that C19orf43 (TRIR) and TNKS act cooperatively to the accumulation of telomeric R-loops formed by the long non-coding RNA TERRA, thereby safeguarding telomere integrity. By limiting persistent RNA–DNA hybrids at chromosome ends, this partnership helps prevent replication interference and aberrant recombination events associated with telomere dysfunction [172]. TNKS2 has been implicated in the Alternative Lengthening of Telomeres (ALT) pathway, a telomerase-independent mechanism exploited by certain cancers to sustain proliferation. Within ALT-positive cells, where elevated TERRA transcription, telomeric DNA damage and recombination converge TNKS2-mediated control of telomeric R-loops may contribute to the regulation of telomere stability. Together, these observations position TRIR–TNKS interactions as a potential modulatory axis in ALT-associated telomere maintenance [172,173].

## 9. Tankyrase, the Proteasome and Protein Degradation

The 26S proteasome comprises a 20S proteolytic core and a 19S regulatory complex which is located at one or both ends of the 20S cylindrical subunit [174,175]. The 19S complex consists of multiple tightly and loosely bound components which function as regulators or cofactors; one such protein is PI31 which has been suggested to be either an activator or an inhibitor of 26S proteasome activity [176,177]. In *Drosophila* TNKS binds directly to DmPI31 and PARylation by TNKS is required for PI31 to be able to increase proteasome activity in *Drosophila* and mammalian cells [178]. Similarly, inhibition of TNKS reduces proteasome activity [178]. Furthermore, in *Drosophila*, PARylation of PI31 by TNKS suppresses its interaction with 20S proteasome α subunits, decreasing the PI31-mediated inhibition of the 20S proteasome [178]. PARylation of DmPI31 enhances its interaction with the 19S dp27 and dS5b chaperone proteins, resulting in their sequestration from 19S, favoring 26S assembly [178]. Using deletion mutants, it has been shown that *Drosophila* TNKS binds DmPI31 through its Ankyrin domain and not through SAM or PARP domains. Similarly, mutation of amino acids in the suggested DmPI31 TNKS binding site (RxxGxGxE/D; actually, RCVGVGDD in DmPI31) negates the interaction with TNKS [178].

Recently it has been shown that TNKS interacts, beside RNF146, with other E3 ligases, specifically the RING-UIM (Ubiquitin-Interacting Motif) family, for example, RNF114 and RNF166 [41]. These proteins bind to the TNKS catalytic SAM/PARP domain, not the usual Ankyrin binding domain. RNF166 promotes K11-linked ubiquitylation of TNKS, requiring TNKS catalytic activity [41]. Significantly, the K11-linked ubiquitylation competes with the well-characterized K48-linked polyubiquitylation and degradation to stabilize TNKS and its binding partners [41]. In a screen of other PAR-dependent E3 ligases that could target TNKS, BAP, CHFR, DTX1, DTX2 and DTX4 were found to co-immunoprecipitate in pull-down experiments. Similar to RNF146, CHFR targets TNKS1 for polyubiquitylation and degradation by the proteasome. Whereas RNF166 induces a diubiquitylated form of the normally monoubiquitylated TNKS, DTX1, 2 and 4 induce stabilization of the monoubiquitylated form with no further ubiquitylation [22]. Another E3 ligase, containing a WWE PAR-binding domain, HUWE1, interacts with TNKS and this is dependent on its catalytic activity. HUWE1 also stimulates TNKS ubiquitylation [41].

Tumor necrosis factor (TNF) can induce cell death through formation of cytosolic complex II, which contains receptor-interacting protein kinase 1 (RIPK1), RIPK3, Fas-associated death domain protein (FADD), TNF receptor associated factor 2 (TRAF2), TNF receptor associated death domain protein (TRADD), receptor interacting protein 1 (RIP1), and caspase-8 reviewed by, for example, ref. [179]. The complex also contains TNKS1 which, in response to a death signal, PARylates components of the complex, causing recruitment of RNF146 and subsequent protein degradation of complex II components, limiting TNF-induced apoptosis [180]. The upstream kinase transforming growth factor-beta-activated kinase 1 (TAK1) normally acts to suppress RIPK1-dependent apoptotic or necroptotic signaling; inhibition or loss of TAK1 unleashes RIPK1’s kinase activity and triggers cell death [181,182]. This places TNKS in the TNF–RIPK1 cell-death axis, connecting upstream TAK1-dependent survival signaling with PAR-modification-driven regulation of complex assembly and execution of RIPK1-dependent pathways.

It has been shown that expression of the SARS-CoV-2 MacroD, like other viral macrodomains, sensitizes cells to TNF-induced cell death [180]. The macrodomain has ADP-ribose hydrolyzing activity and sensitizes cells to TNF-induced cell death by abolishing PARylation of complex II components [180].

Several other TNKS binding proteins have been characterized, with their interaction serving as a precursor to PARylation and subsequent degradation. The range of targets for TNKS is notably diverse, and as a result, the downstream effects within the cell are equally varied and complex.

Centrosomal P4.1-associated protein (CPAP), a protein involved in centrosome function, is PARylated in vivo and in vitro by TNKS [183]. Interaction is through a very highly conserved TNKS binding motif (REYPDG) located in the C-terminal G-Box of CPAP. Over-expression of TNKS leads to ubiquitylation and proteasome-mediated degradation of CPAP, preventing centriole duplication [183]. Reduction in TNKS expression, causing increased CPAP level, results in aberrant centrioles and multipolarity. It has been concluded that TNKS localizes to centrosomes promoting degradation of CPAP in early G1. The histone methyltransferase EZH2 regulates transcription of TNKS and thus EZH2-mediated TNKS suppression reduces CPAP causing centrosome overduplication and multipolar mitosis [184].

Interestingly, in a study of *Drosophila* development it was observed that *Drosophila* JNK (Bsk) serves as a substrate for TNKS-mediated PARylation, followed by ubiquitylation [185]. Thus, TNKS can affect lifespan, stress tolerance, and energy homeostasis through subtle regulation of Bsk. PARylation by TNKS leads to K63-linked poly-ubiquitylation on JNK, enhancing its kinase activity and maintaining its in vivo functions [155,185]. The K63-linked modification does not lead to proteasome-mediated degradation but probably acts to affect Bsk secondary structure and enzyme activity [185]. Other studies have shown that activation of *Drosophila* JNK by TNKS has a role in regulating apoptosis [146].

## 10. Tankyrases and Glucose Metabolism

TNKS activity is associated with the regulation of glucose metabolism in mammals [186]. Furthermore, the TNKS inhibitor G007-LK causes a reduction in PARylation and subsequently improves systemic glucose tolerance in adipocytes in female mice [186]. The glucose transporter, GLUT4, regulates the insulin-mediated transport of glucose in fat and muscle cells [187]. In the absence of insulin, GLUT4 is sequestered in GLUT4 storage vesicles (GSVs), whereas binding of insulin to its receptor causes release of GLUT4 to the cell surface. Ubiquitylation is necessary for transport of GLUT4 to the GSVs; this is followed by deubiquitylation, preventing degradation [188]. Like GLUT4 the insulin-responsive aminopeptidase IRAP localizes within GSVs and translocates to membranes in response to insulin [189]. IRAP interacts with TNKS through a TNKS binding motif (RQSPDG), comprising residues 96–101 in the IRAP cytosolic domain [38]. The deubiquitylating enzyme USP25 binds to the ankyrin repeats of TNKS through a TNKS binding motif (RTPADG) located at residues 1049–1054 [71]. Significantly, USP25, TNKS, and GLUT4 colocalize in insulin-sensitive cells. Depletion of USP25 with siRNA causes a reduction in GLUT4 level in adipocytes and this leads to a loss of insulin’s ability to stimulate glucose transport [190]. Although TNKS plays an important role in the regulation of GLUT4 level it has also been demonstrated that its inhibition results in downregulation of other GSV proteins, RAB10, VAMP8, and SORT1, although in that study it was not confirmed that they interact with TNKS [191]. Thus, it has been concluded that TNKS binding and ubiquitylation of IRAP and other GSV proteins and association with USP25 controls glucose transport in adipocytes and muscle cells [190].

Beyond its role in GLUT4 vesicle dynamics, TNKS also regulates glucose metabolism at the enzymatic level through its interaction with GDP-mannose 4,6-dehydratase (GMD), a key enzyme in the GDP-fucose biosynthetic pathway. GMD contains a functional TNKS -binding motif; however, in vitro PARP assays and in vivo analyses showed that GMD is unable to be PARylated by TNKS [46]. In fact, the binding of GMD to TNKS suppresses its catalytic PARP activity and this inhibition is also specific for TNKS and not for other PARPs like PARP1, since it requires the presence of the TBM in GMD [46,192]. As GDP-fucose availability influences glycosylation of membrane proteins and receptors, including those involved in insulin signaling, this TNKS-GMD regulatory axis provides an additional metabolic mechanism through which TNKS modulates glucose homeostasis. Collectively, these findings suggest that TNKS coordinates both vesicular transport and glycosylation-linked metabolic pathways to fine-tune glucose uptake and energy balance in mammalian cells.

## 11. Tankyrase as a Central Regulator of Cellular Signaling Networks

Apart from their roles in Wnt/β-catenin and DNA damage repair pathways, and the additional signaling networks discussed earlier in this review, TNKS enzymes function as multifaceted scaffolds that coordinate several other major signaling cascades. They do so by regulating the stability and activity of key adaptor proteins through PARylation-dependent ubiquitylation. In metabolic regulation, TNKS associates with the serine/threonine kinase LKB1 (STK11) via a conserved TNKS-binding motif, promoting PARylation of LKB1 glutamate residues and subsequently its RNF146-mediated ubiquitylation, thereby limiting LKB1–AMPK activation and enhancing mTORC1 signaling [7]. Conversely, TNKS inhibition stabilizes LKB1, leading to AMPK activation and metabolic reprogramming. In the PTEN–Akt axis, it has been suggested that TNKS interacts with PTEN, and PARylation of PTEN promotes its proteasomal degradation, indirectly sustaining Akt activity and cell proliferation [193]. However, recent studies have cast doubt on these observations and it has been concluded that the proposed TBM does not, in fact, bind to TNKS ARCs, PTEN is not PARylated, and TNKS does not associate with PTEN in vitro [194]. Within the SRC/immune signaling network, TNKS regulates the adaptor SH3BP2 (3BP2), a positive modulator of SRC and SYK kinases in immune cells. PARylation of 3BP2 by TNKS1/2 triggers its RNF146-dependent ubiquitylation and turnover, thereby restraining SRC-mediated immune and inflammatory signaling [8]. Cherubism is an autosomal dominant autoinflammatory disorder associated with loss of bone in the jaw and accumulation of fibrous tissue in the face, temporarily giving rise to chubby cheeked infants. It is caused by mutations in the SH3 binding protein SH3BP2, such that it no longer interacts with TNKS [8,9,195]. Mutations in the TNKS binding motif (RSPPDG) located at residues 413–418, between the PH and SH2 domains of SH3BP2, resulting in ablation of PARylation of SH3BP2 by TNKS and subsequent ubiquitylation by RNF146 [8,9]. Overexpression of SH3BP2 causes hyperactivation of the SRC, SYK, and VAV signaling pathways, with effects on bone dynamics, metabolism, and Toll-like receptor (TLR) signaling reviewed by [40,196]. In the YAP/Hippo pathway, TNKS controls the fate of the angiomotin (AMOT) family proteins, AMOT, AMOTL1, and AMOTL2, which function as scaffolds linking polarity and Hippo components. TNKSs interact with AMOT family proteins, binding to classical TBDs [67,197]. PARylation of AMOTs is followed by ubiquitylation by RNF146. Ubiquitinated AMOTs are degraded by the proteasome [67]. In addition, PARylated AMOTS are also recognized by a second E3 ligase, RNF166, which binds through a novel C-terminal Di19-ZF domain [198]. PARylation of AMOTs also reduces their ability to sequester YAP/TAZ in the cytoplasm, thereby promoting YAP nuclear localization and transcriptional activation [199]. TNKS has recently been implicated in the regulation of miRNA biogenesis through its interaction with the Microprocessor complex [200]. DGCR8, the RNA-binding scaffold of the Microprocessor, cooperates with Drosha to cleave primary miRNA transcripts (pri-miRNAs) and initiate canonical miRNA maturation. Proteomic and biochemical studies have shown that TNKS1/2 interact with the DGCR8–Drosha complex via their ankyrin-repeat clusters (ARCs), enhancing Microprocessor activity and promoting more efficient processing of specific pri-miRNAs [200]. Although the authors identified candidate TBMs within DGCR8 and Drosha, these linear motifs have not yet been structurally validated as canonical TBM–ARC interfaces. Nevertheless, TNKS inhibition reduces pri-miRNA cleavage efficiency, supporting a model in which TNKS functions as a positive modulator of Microprocessor assembly or stability [200]. These findings broaden the functional scope of TNKS beyond its well-defined roles in Wnt signaling and telomere regulation, positioning TNKS as an upstream regulator of miRNA biogenesis [200].

CD2-associated protein (CD2AP), a scaffolding adaptor involved in cytoskeletal organization and membrane dynamics, forms a functional interaction with TNKS1/2 and acts as a negative regulator of their activity. In podocytes, loss of CD2AP leads to unchecked TNKS function, resulting in heightened β-catenin activation and increased global PARylation. This dysregulated TNKS–CD2AP axis is closely associated with podocyte injury, highlighting CD2AP as a critical modulator linking adhesion, signaling, and cytoskeletal integrity within the glomerular filtration barrier [201].

FBP17, a membrane-curving F-BAR adaptor protein involved in endocytosis and actin remodeling, forms a functional association with TNKSs through a conserved TBM that enables transient, signal-responsive docking onto TNKS ARCs [202]. Through this dynamic interaction, which probably couples brief TNKS engagement with PARylation-dependent modulation of FBP17 turnover, TNKSs are integrated into membrane deformation and trafficking processes [202]. Dysregulation of this FBP17–TNKS axis alters local PAR signaling and cytoskeletal organization, highlighting FBP17 as an important spatial coordinator of TNKS activity in actin- and membrane-dependent cellular events [202].

GRB14 is a member of a small family of adaptor proteins that interact with multiple receptor tyrosine kinases and signaling molecules, including the insulin and insulin-like growth-factor receptors. It binds the ankyrin-repeat region of TNKS2 through its N-terminal 110 amino acids, forming a physical association enriched in low-density microsomal fractions. This interaction promotes PARylation-dependent turnover of GRB14, revealing a previously unrecognized role for TNKS in adaptor-protein-mediated signal transduction and vesicle-trafficking pathways, extending their function beyond telomere maintenance and mitotic regulation. Through this mechanism, GRB14 may modulate insulin receptor signaling by influencing site-specific tyrosine dephosphorylation, thereby integrating TNKS activity into the fine control of metabolic signaling [203].

Regulation of mRNA turnover is increasingly recognized as a dynamic layer of post-transcriptional control that integrates signaling pathways with gene expression programs. TNKS1 contributes to this regulation by modulating RNA-binding protein activity, exemplified by its interaction with HuR, which stabilizes specific transcripts to support muscle fiber formation. Beyond this, TNKS-associated factors extend their influence into broader mRNA decay pathways [204]. As mentioned earlier, TAB182 serves as an interchangeable cofactor of the CCR4–NOT complex, a central effector of mRNA turnover that initiates decay through 3′ poly(A)-tail shortening [88,89]. Through TAB182’s association with CCR4–NOT, TNKS-linked functions converge with this major deadenylase machinery, suggesting coordinated control over transcript stability and degradation. Notably, core CCR4–NOT subunits CNOT2 and CNOT3 also act as positive modulators of Wnt/β-catenin signaling [90] and emerging research has shown that the CCR4–NOT complex plays an important regulatory role in genome stability and the DNA damage response signaling pathway [205,206], highlighting additional layers of crosstalk in which mRNA turnover machinery intersects with canonical signaling pathways to fine-tune cellular responses.

## 12. Tankyrase Involvement in Disease and Tankyrase Inhibitors

Collectively, the findings outlined in this review highlight TNKS as a central post-translational regulator that integrates energy metabolism, growth, immune signaling, and mechanotransduction through context-dependent control of its diverse substrate network. TNKS provides a mechanistic link between signaling and genome maintenance: its canonical role in Wnt/β-catenin control via AXIN degradation intersects with DDR pathways, as Wnt activity modulates the expression of repair genes, while oxidative stress and DNA damage feed back onto Wnt-signaling [207]. Furthermore, the importance of TNKS in pathways as diverse as glucose metabolism and telomere maintenance highlights its significance in maintaining cellular function and overall organism well-being. Therefore, it is to be expected that malfunction of the TNKS-dependent pathways will have severe clinical consequences. Dysregulation of the Wnt signaling pathway plays an important role in tumorigenesis, cancer progression, metastasis, invasion, as well as in therapeutic resistance [208,209,210,211]. Historically, initial observations established that mutations in the APC gene were responsible for the hereditary colon cancer syndrome, familial adenomatous polyposis. APC interacts with β-catenin and loss of APC activity leads to unrestricted β-catenin signaling, establishing links between Wnt signaling complex and colorectal cancer [209,211,212]. Wnt signaling is also activated in other cancers, such as breast cancer, other forms of gastrointestinal cancers, melanoma, and leukemias [210,212,213]. It appears that Wnt-associated signaling can initiate tumor development in various leukemia subtypes, such as acute lymphoblastic leukemia and chronic lymphocytic leukemia [213]. Disruption in Wnt signaling also contributes to the onset and progression of other diseases, such as cardiovascular disease, neurodegenerative disease, metabolic disorders and autoimmune disease [78,212,213,214,215,216]. Specific aspects of cardiovascular disease affected include atherosclerosis, myocardial infarction and arrhythmogenesis [217,218]. Wnt signaling has been linked to the neurodegenerative diseases ALS, Alzheimer’s disease, and Parkinson’s disease [213,219]. As the Wnt pathway regulates various aspects of metabolism including lipid metabolism, insulin signaling, and glucose homeostasis it is to be expected that it will impact on metabolic diseases such as Type 2 diabetes mellitus (T2DM) and obesity [213,220,221]. Whilst there is little evidence to suggest that mutations in the *TNKS* genes are responsible for dysregulation of the Wnt signaling pathways it has become apparent that small molecule TNKS inhibitors offer an effective means of inhibiting the Wnt pathway, with considerable therapeutic potential [24,222,223].

Defects in DNA repair pathways are well-established as the causes of cancers and neurological and immunological defects [224,225]. Because TNKSs play an important part in DSB repair, with roles in HR and NHEJ, dysregulation of their activities could contribute to genomic instability and subsequent disease. Tankyrases contribute to telomere maintenance directly through PARylation of TRF1, enabling telomerase engagement, and indirectly through their influence on Wnt signaling, which supports telomere length preservation during stem cell division. Telomere dysfunction tends to amplify the normal hallmarks of aging, possibly increasing the likelihood of age-related diseases such as cancer and neurodegeneration. As mentioned in Section 8, TNKSs also contribute to the maintenance of telomeres by favoring the repair of damaged telomeric DNA. However, there appears to be little evidence to suggest that telomere dysfunction is directly attributable to tankyrase mutation, although aberration in Wnt signaling will have effects on telomere maintenance. Whether this represents a possible opportunity for intervention through tankyrase inhibitors will have to await future investigation.

The involvement of tankyrases in glucose metabolism is partially via the Wnt pathway but importantly through the regulation of GLUT4 dynamics and the level of other GSV proteins and by interaction with GMD, as described in Section 10. Thus, TNKSs have significant direct and indirect roles in metabolic diseases like obesity and T2DM. It is possible that tankyrase inhibitors could be used to improve insulin sensitivity and reduce fat accumulation as a possible clinical intervention although this is unlikely to be as effective as the glucagon-like peptide-1 (GLP-1) receptor agonist, semaglutide, which is now in common use.

Two principal classes of TNKS inhibitors are recognized: inhibitors that bind the nicotinamide subsite, such as XAV939, RK582, and others that stabilize AXIN, and those that bind the adenosine subsite, including IWR-1, G007-LK, and K-756 [23,24]. Similarly, in the treatment of neuroblastoma, XAV939 has been shown to inhibit the Wnt/β-catenin signaling pathway and reduce the expression of anti-apoptotic proteins [150]. TNKS inhibitors have been combined with other agents, such as PIK3 inhibitors to improve responses in APC-mutant colorectal cancer [226,227]. XAV939 has also been used to enhance radio- and chemosensitivity in the treatment of, for example, colon and cervical cancers [228,229].

TNKS inhibitors face significant selectivity challenges because the TNKS catalytic (ART) domain shares substantial homology with the catalytic cores of PARP1 and PARP2, particularly within the conserved NAD^+^-binding pocket, raising the risk of off-target PARP1/2 inhibition and consequent impairment of single-strand break repair [23,53]. Disruption of PARP1/2 activity in this context can contribute to genotoxicity [24,230]. In addition, TNKS inhibition interferes with mitotic spindle assembly via loss of NuMA PARylation [4,36,64,137,183].

These effects can induce toxicity in normal proliferating cells. Although exploiting TNKS-specific structural features, such as the CHCC zinc-binding motif or unique elements of the TNKS D-loop, could theoretically improve selectivity, the highly conserved architecture of PARP catalytic domains limits the feasibility of this approach [52,53,54]. Collectively, the extensive interdependence between TNKS-regulated signaling pathways and DNA damage response systems explains why TNKS inhibitors, despite therapeutic potential, can produce DDR-related on-target and off-target effects, necessitating careful patient stratification, rational combination strategies, and cautious clinical deployment [107,231].

## 13. Concluding Remarks

TNKS1/2 have evolved from being viewed as niche regulators of telomeres and Wnt signaling to multifunctional architects of cellular organization. Their modular design combining selective ARC–TBM recognition, dynamic PARylation kinetics, and scaffolding capacity enables TNKS1/2 to reshape protein networks across compartments ranging from centrosomes and damage foci to peroxisomes and innate immune hubs. This versatility places TNKS at the intersection of genome stability, signal transduction, metabolism, and cell fate control, where they operate less as linear enzymes and more as higher order organizers of molecular circuits.

The emerging picture is that TNKS-dependent PARylation does not simply tag substrates for destruction but encodes a broader biochemical language that dictates whether proteins are degraded, remodeled, stabilized, or compartmentalized. This PAR-centered coding, integrated with ubiquitin signaling and kinase networks, allows TNKSs to tune pathway thresholds with exceptional precision, a property now recognized in contexts as diverse as tumorigenesis, viral pathogenesis, neuronal resilience, and metabolic adaptation.

As the TNKS interactome continues to expand, a major challenge will be to define how combinatorial TBM classes, PAR chain architectures, and subcellular microenvironments together generate pathway-specific outcomes. Equally pressing is the need to disentangle catalytic from structural roles, given that TNKS function as both writers of PAR codes and scaffolds for macromolecular assemblies. These questions are central to the development of next-generation TNKS inhibitors capable of pathway-selective modulation rather than global suppression. Achieving this precision will be crucial for safely exploiting TNKS as therapeutic entry points in Wnt-driven cancers, DDR-defective tumors, metabolic disease, and viral infection.

In summary, TNKS represents a paradigm in which a single enzyme family acts as a systems-level regulator, linking genome surveillance, signaling fidelity, and metabolic control. Continued structural, computational, and mechanistic studies will be essential to fully decode TNKS biology and harness their potential as strategic nodes for therapeutic intervention.

## Data Availability

No new data were created or analyzed in this study.

## Supplementary Materials

The following supporting information can be downloaded at: https://www.mdpi.com/article/10.3390/cells15040348/s1, Table S1: Tankyrases and their binding proteins.

## Abbreviations

Akt	Protein kinase B
ALS	Amyotrophic Lateral Sclerosis
ALT	Alternative Lengthening of Telomeres
AMOT	Angiomotin
AMOTL	Angiomotin-like
AMPK	AMP-associated kinase
APC	Adenomatous polyposis coli gene/protein
ARC	Ankyrin repeat cluster
ARD	Ankyrin repeat domain
ARTD	ADP-ribosyltransferase domain
ARH3	ADP-ribosylhydrolase 3
ART	ADP-ribosyltransferase
ATG	Autophagy related gene
BAP	BRCA1-associated protein
Bcl2	B-cell lymphoma 2
Β-TrCP	Beta-transducin repeat-containing protein
BLM	Bloom syndrome protein
BRCA1	Breast cancer gene 1
BRISC	Brcc36 isopeptidase complex
CCR4-NOT	Carbon catabolite repression 4 (Ccr4)-negative on TATA-less
CD2AP	CD2-associated protein
CHFR	Checkpoint with forkhead and ring finger domains
CK1	Casein kinase 1
CNOT	(Ccr4)-negative on TATA-less
CPAP	Centrosome Assembly and Centriole Elongation Protein
CRC	Colorectal cancer
CtIP	C-terminal interacting protein binding protein
DDR	DNA damage response
DGCR8	DiGeorge syndrome critical region 8
DIAP1	Death-associated inhibitor of apoptosis 1
DNA-PKcs	DNA dependent protein kinase catalytic subunit
DS	Dyad symmetry
DSB	Double strand break
DTX	Deltex protein
DUB	Deubiquitinase
DVL	Dishevelled gene
E2F	E2 promotor binding factor
EBV	Epstein–Barr virus
ESCC	Esophageal squamous cell carcinoma
FADD	Fas-associated death domain protein
FBP17	Formin binding protein 17
FHL2	Four-and-a-half LIM-only protein 2
FTD	Frontotemporal Dementia
FUS	Fused in Sarcoma
GLUT4	Glucose uptake 4
GMD	GDP-mannose 4,6-dehydratase
GRB14	Growth factor receptor-bound protein 14
GSK3	Glycogen synthase kinase-3
GSV	GLUT4 storage vesicles
HCC	Hepatocellular carcinoma
HH4	Human herpesvirus 4
HR	Homologous recombination
HSV-1	Herpes simplex virus-1
HuR	Human antigen R
IAP	Inhibitor of apoptosis
IAV	Influenza A virus
ICP0	Infected cell protein 0
IFNB1	Interferon β1
IL6	Interleukin 6
ISG56	Interferon stimulated gene 56
JNK	c-Jun N-terminal kinase
LKB1	Liver kinase B1
LLPS	Liquid–liquid phase separation
MAPK	Mitogen-activated protein kinase
MAR	Mono(ADP-ribose)
MAVS	Mitochondrial antiviral signaling protein
Mcl-1	Myeloid cell leukaemia 1
MDC1	Mediator of DNA Damage Checkpoint 1
MERIT40	Mediator of RAP80 Interaction and Targeting 40
MND	Motor Neuron Disease
mTORC1	Mammalian target of rapamycin complex 1
NHEJ	Non-homologous end joining
NKD	Natural killer cell deficiency protein
NLS	Nuclear localization signal
NuMA	Nuclear Mitotic Apparatus Protein 1
OriP	Origin of replication P
PAR	Poly(ADP-ribose)
PARG	Poly (ADP-ribose) glycohydrolase
PARP	Poly(ADP-ribose) polymerase
PCP	Planar cell polarity
PEX	Pexophagy
PI31	Proteasomal Inhibitor of 31kD
Plk1	Polo-like kinase 1
POT1	Protection of telomere 1
PP1	Protein phosphatase 1
PTEN	Phosphatase and tensin homologue
RAP1	Repressor/Activator Protein 1 homologue
RAP80	Receptor-associated protein 80
Rb	Retinoblastoma protein
RBP	RNA-binding protein
RIP1	Receptor interacting protein 1
RIPK1	Receptor-interacting protein kinase 1
RNF	Ring finger protein
SA1	Cohesin subunit 1
SAM	Sterile alpha motif
SARS-CoV-2	Severe acute respiratory syndrome coronavirus 2
SeV	Sendai virus
SH3BP2	SH3 domain binding protein 2
SRC	Sarcoma proto-oncogene
SSB	Single strand break
T2DM	Type 2 diabetes mellitus
TAB182	Tankyrase 1 binding protein 1 of 182kDa
TAK1	TGFβ-activated kinase 1
TBM	Tankyrase binding motif
TCF/LEF	T cell factor/lymphoid enhancer factor
TDP-43	Transactive response DNA binding protein of 43KDa
TERRA	Telomeric repeat containing RNA
TIN2	TERF-interacting nuclear factor 2
TLR	Toll-like receptor
TNKS	Tankyrase
TNKS1	Tankyrase 1
TNKS2	Tankyrase 2
TRADD	TNF receptor-associated death domain protein
TRAF2	TNF receptor-associated factor 2
TRF1	Telomeric repeat factor 1
TRIR	Telomerase RNA Component Interacting RNase
UIM	Ubiquitin-Interacting Motif
USP25	Ubiquitin specific protease 25
UTR	Untranslated region
VISA	Virus-induced signal adaptor
Wnt	Wingless and Int-1
WRN	Werner syndrome protein
XRCC4	X-ray repair cross-complementing group 4
YAP	Yes-associated protein 1
